# Progress and Insights in the Application of MXenes as New 2D Nano-Materials Suitable for Biosensors and Biofuel Cell Design

**DOI:** 10.3390/ijms21239224

**Published:** 2020-12-03

**Authors:** Simonas Ramanavicius, Arunas Ramanavicius

**Affiliations:** 1 Center for Physical Sciences and Technology (FTMC), Sauletekio av. 3, LT-10257 Vilnius, Lithuania; simonas.ramanavicius@ftmc.lt; 2 Institute of Chemistry, Department of Physical Chemistry, Faculty of Chemistry and Geosciences, Vilnius University, Naugarduko 24, LT-03225 Vilnius, Lithuania

**Keywords:** MXenes, 2D-nanoparticles, 2D-nanomaterials, catalytic electrochemical biosensors, redox enzymes, nonstoichiometric titanium oxides TiO_2−x_/TiO_2_ and Ti_n_O_2n−1_, immunosensors, antibodies, enzymatic biofuel cells, microbial biofuel cells, bioelectrochemistry

## Abstract

Recent progress in the application of new 2D-materials—MXenes—in the design of biosensors, biofuel cells and bioelectronics is overviewed and some advances in this area are foreseen. Recent developments in the formation of a relatively new class of 2D metallically conducting MXenes opens a new avenue for the design of conducting composites with metallic conductivity and advanced sensing properties. Advantageous properties of MXenes suitable for biosensing applications are discussed. Frontiers and new insights in the area of application of MXenes in sensorics, biosensorics and in the design of some wearable electronic devices are outlined. Some disadvantages and challenges in the application of MXene based structures are critically discussed.

## 1. Introduction

**Figure 1:**
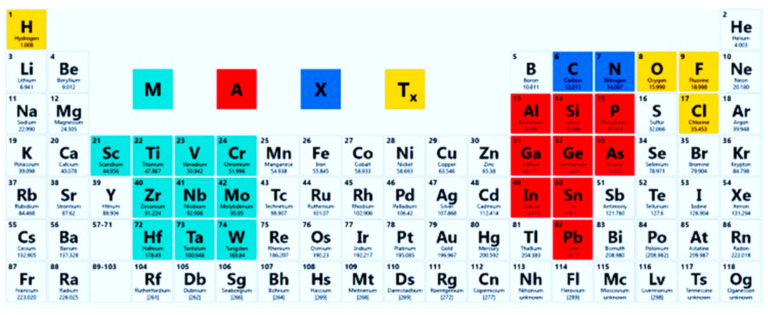
The composition of MXenes and MAX phases from the periodic table. Reprinted from [12].

MXenes have appeared very recently (in 2011) as a new class of 2D materials with either metallic conductivity [1,2], or some attractive semiconducting properties, or both, which can be well exploited in the design of sensors, biosensors, biofuel cells and in the development of some wearable bioelectronic devices. MXenes have some structural relation and even similarity of some physical properties with other 2D materials such as graphene [3,4]. Most MXenes are based on 2D transition metal carbides [2]. The most of MXenes are based on 2D transition metal nitrides carbonitrides are appointed to this class of MXene materials [5]. MXenes are usually prepared by etching of initial materials, called “MAX phases”, which can be presented by generalized formula M_n+1_AX_n_ in which “M” representing the transition metals (that are Ti, Sc, Zr, Cr, V, Mn, Hf, Nb, Mo or Ta), “A” is an element from group 12, 13, 14, 15 or 16 (that are Al, Cd, Si, S, P, Ga, As, Ge, In, Tl, Sn or Pb) in the periodic table, “X” is either carbon (C), nitrogen (N) or a mixture of both of them [6,7,8,9], and “n” in this formula can be in the range of 1–3 [6,7,9,10,11] (Figure 1).

It should be noted that MAX phases are characterized by metal-like electrical/thermal conductivity behavior and they are mostly chemically stable materials. MXenes possess great and rather unusual physical and chemical properties that can be well adapted for the design of electrochemical sensors and biosensors. The properties of MXenes can be well tailored through proper variation of M and X elements in MXene structure and by the introduction of various surface terminal groups [13,14,15]. Due to this option of applying very different surface “finishing”, recent advances in surface chemistry enables the introduction of particular surface “terminal functional groups” [13,14,15], which can be suitable for the immobilization of enzymes and some other proteins. Hence, MXenes can be efficiently modified by particular biomolecules and many other compounds that are required for the action of biosensors. In addition, the above mentioned “terminal functional groups” can provide tailored electronic, electrochemical and optical properties to MXene-based biosensing structures [13,14,15]. Optical properties of MXenes are highly applicable for biosensing purposes [16], especially those which are based on fluorescence resonance energy transfer and induce changes in photoluminescence signal [17]; however, the applicability of MXenes in optical biosensors is well reported in specialized review [18], therefore, in this paper we are not aiming to address many details of optical MXene-based biosensors. In some researches [19,20,21,22,23,24,25,26,27] and reviews [18,28] it is reported, that MXenes are compatible with enzymes and other protein molecules, which are used in the development of catalytic biosensors and affinity sensors. Biocompatibility of MXenes towards some microorganisms [29,30] and even towards mammalian neural cells [31], was also determined. Catalytic activity has been reported for some MXenes, but immobilized enzymes and microorganisms can significantly extend the ability to utilize significantly broader ranges of substrates, which can provide chemical energy for biofuel cells. Bioelectronics devices dependent on the type of applied bio-recognition elements can be divided into several classes, such as: (i) catalytic sensors and biofuel cells based on enzymes [32] and non-enzymatic structures [33], (ii) whole-cell-based biosensors and biofuel cells [34], (iii) affinity sensors based on immobilized antibodies or antigens (immunosensors) [35], (iv) immobilized single stranded DNA (ssDNA)-based sensors (DNA-sensors) [36], molecularly imprinted polymer-based sensors, [37] et cetera. Applicability of various nanomaterials in some of these classes of biosensors has been demonstrated [38]. Some researchers predicted that MXenes will form the basis for various MXene nanocomposites and development of commercially available MXene-based biosensors [39]. Therefore, in this review we are addressing recent advances in the applicability of MXenes in these areas and are predicting future developments in this expanding area.

The aim of this review is to present insights for the applicability of MXenes in the design of biosensors and biofuel cells. A very attractive property of biofuel cells is that they operate at room temperature and are capable of producing electricity from highly diluted solutions of chemical fuels.

## 2. Catalytic Sensors Based on Enzymes

Catalytic sensors based on enzymes and some other redox proteins have several advantages over other analytical systems because they can provide high selectivity. One of the mostly expanded areas of enzymatic sensors is related to the development of electrochemical sensors and advantages of such sensors are based on low costs, simple operation and ability to being applied for the evaluation of optically badly transparent and turbid samples, for example, blood. Moreover, recently implantable biosensors are appearing in the market and these require biocompatible materials for the design of electrodes and biofuel cells, which will supply power for these implantable devices. Therefore, efficient charge transfer between electrodes and immobilized enzymes is very important issues [40,41]. Moreover, sometimes it is possible to establish direct charge transfer (DCT), which is sometimes called direct electron transfer but this fact is not always correct, because our recent researches show that sometimes DCT is established between p-type semiconducting polymers and redox enzyme, glucose oxidase (GOx) [42,43]. The establishment of direct charge transfer (DCT) between redox enzymes enables exploiting of inherent thermodynamic potential of the enzyme-catalyzed reaction in the design of electrochemical-catalytic biosensors and all types of biofuel cells, which in the most optimal cases are free of soluble redox mediators [40,41,44,45,46]. Cytochromes can be applied in the design of enzymatic sensors and biofuel cells as catalysts and as redox mediators that are capable of establishing DCT between enzymes and electrodes [32]; and due to this charge-transfer based interaction they can change the optical properties of either Ti_3_C_2_ MXene based ultra-thin nanosheets, quantum dots, or both, which were applied in the design of biosensors dedicated to the determination of human papilloma virus [47] and trypsin [48]. Charge transfer property was also applied in the design of glucose sensors, where titanium carbide based MXenes were combined with red-emitting carbon dots [49]. Hence, the application of MXenes in DCT-based systems is rather promising; the Nafion stabilized Ti_3_C_2_T_x_-based MXene (where T_x_ was some transition metal) was applied for sensing of dopamine [50], and electrochemical characterization of glassy carbon electrode (GCE) modified by Nafion (Nafion/Ti_3_C_2_T_x_/GCE) revealed large surface area, large intrinsic conductivity and low charge transfer resistance. Therefore, it was predicted that MXenes can be well implemented into the construction of enzymatic sensors and biosensors and probably direct charge transfer between MXenes and some redox enzymes can be established. However, establishment of DCT between redox enzymes and solid electrodes is rarely possible, because redox-active sites of most enzymes are rather deeply encapsulated within “electrically insulating” protein structures, which have low electric permittivity (*ε*) [51,52]. Therefore, various soluble redox mediators are applied in the design of such biosensors [53] and biofuel cells [32]. However, soluble redox mediator based strategies are not very efficient, therefore, some conducting nanomaterials including carbon nanotubes [54], gold nanoparticles [44], conducting polymers [55] and very recently, MXenes, can be applied for the purpose of either establishing direct charge transfer between redox enzymes and electrode surfaces, facilitating charge transfer from products formed during enzymatic reaction [56,57,58,59], or both. An even more complicated situation is charge transfer from microorganisms because most of them are wrapped within an “electrically insulating” layer of polysaccharides [60,61], but recently we have found how conductivity of cell-walls can be improved by the formation of conducting polymer nanoparticles [60,62] and even some larger structures [61] within the cell wall of microorganisms, and in such a way the conductivity of this initially nonconducting structure can be remarkably improved [34,63]. This formation of conducting polymers can be induced by the metabolic cycle of these microorganisms [64] and conducting polymer-based structures are well distributed within either cell wall, in the periplasm of microorganism [62,65], or both.

Ti_3_C_2_-based MXene was used for the immobilization of tyrosinase within the pre-adsorbed chitosan (CS) layer and Tyr/MXene/CS/GCE was applied for the determination of phenol in water with sensitivity of 414.4 mA M, linear range between 0.05–15.5 mM and with LOD of 12 nM [21]. Drop-casting based dispersion was performed and electrostatic effects between MXene and tyrosinase enabled proper orientation of the enzyme during the immobilization and preserved catalytic activity of immobilized tyrosinase; therefore, very efficient direct charge transfer between tyrosinase and the electrode has been established [19,66].

It should be noted that not only electrons, but holes, can be involved in direct charge transfer within redox enzyme structure and at interphase between enzyme and electrode [42,43], therefore, MXenes in this charge-transfer related context are especially interesting, because they can act as metallic conductors with varied conductivity, which depends on applied “M” and “X” elements in the structure of MXenes (M_n+1_X_n_T_x_) and covalent surface modifications in their structure [67]. It is remarkable that covalent surface modifications in the structure of MXenes enable achieving superconductivity [67]. The redox-ability, in addition to good conductivity, enables MXenes to facilitate electrochemical redox processes [68]. Hence, some attempts to demonstrate the applicability of MXenes in the design of redox mediator free sensors was demonstrated [19,31] and discussed [5,69], which promises great applicability for MXenes to be applied in various bioelectronics devices including biosensors and biofuel cells. It was demonstrated that if some redox enzymes are trapped within MXene sheets, then rather efficient charge transfer from the active site of enzyme towards electrode can be achieved due to the sufficient mobility of charge within MXene-based structure [70]. In this way β-hydroxybutyrate dehydrogenase decorated MXene nano-sheets were applied for the amperometric determination of β-hydroxybutyrate [71].

MXene and platinum nanoparticle (PtNPs) based nanocomposite was developed and deposited on GCE electrode (Ti_3_C_2_T/PtNPs/GCE) [27], PtNPs significantly enhanced electro-catalytic activity of this MXene-based electrode, and it was sensitive to various compounds that are important during the development of biosensors and biofuel cells, including dopamine, ascorbic acid, uric acid, acetaminophen and H_2_O_2_. Such advantageous sensitivity of Ti_3_C_2_T/PtNPs/GCE electrodes can be potentially adapted for the design of biofuel cell cathodes.

An electrochemical biosensor based on MXene/DNA/Pd/Pt/GCE electrode was developed and applied for amperometric determination of dopamine in the range between 0.2 and 1000 mM with LOD of 30 nM [72]. In this research DNA was important for the dispersion of Ti_3_C_2_-based nano-sheets and formation of Pd and Pd/Pt structures, while Ti_3_C_2_-based MXene acted as a conducting support. However, this MXene/DNA/Pd/Pt/GCE structure was highly sensitive to glucose, uric acid and ascorbic acid. Therefore, this structure is probably better suited for development of biofuel cells, which will be able to consume much broader ranges of biological fuels.

In many researches it was demonstrated that if electrochemically active surface areas of electrode are not sufficient, then a decoration by metal nanoparticles [52,56,58] or some other nanoparticles can be applied in order to increase catalytic activity and thus the currents registered by electrodes modified by enzymes. A similar strategy was applied during the development of some MXene-based catalytic sensors [31,73]. Ti_3_C_2_-based MXene modified by gold nanoparticles (AuNPs) was applied for the Nafion-based immobilization of glucose oxidase (GOx) in order to design a biosensor for the determination of glucose, which was based on Nafion/GOx/AuNPs/Ti_3_C_2_/GCE structure [31]. It was predicted that in this structure, AuNPs are involved in charge transfer between the catalytic site of the enzyme and electrode. In another research, GOx was entrapped within three-dimensional porous Ti_3_C_2_T_x_ MXene, graphene hybrid films, and also applied for glucose determination [74].

Amperometric biosensor based on acetylcholinesterase (AChE) immobilized on Ti_3_C_2_T_x_ and chitosan (CS) modified glassy carbon electrode (AChE/CS/Ti_3_C_2_T_x_/GCE) has been developed and applied for the determination of organophosphorus pesticide—malathion [26]. CS/Ti_3_C_2_T_x_-based heterostructures provided great environments for the immobilized AChE. A similar AChE/CS/Ti_3_C_2_T_x_/GCE-heterostructure was applied for the determination of malathion in tap water [73], while acetylcholinesterase (AChE) immobilized on Ti_3_C_2_T_x_ modified by silver nanoparticles (AgNPs) was developed and also applied for differential pulse voltammetry (DPV)-based determination of organophosphate pesticide – malathion [75]. In this sensor, negatively charged acetylcholinesterase was electrostatically attracted to the surface of positively charged Ag/Ti_3_C_2_T_x_-composite, which improved charge transfer from acetylcholinesterase.

Electrochemical biosensors based on acetylcholinesterase, MnO_2_/Mn_3_O_4_ micro-cuboids, AuNPs and MXenes (AChE/CS/Ti_3_C_2_/AuNPs/MnO_2_/Mn_3_O_4_/GCE) were developed for the determination of some organophosphate—methamidophos, which has acted as inhibitor of immobilized enzyme—AchE [76]. Some researchers are reporting that anodic potentials, which are exceeding +200 mV, lead to the oxidation of its outer surface of Ti_3_C_2_T-based MXenes and can be applied for the oxidation of NADH [20]; this remarkable finding is eternally important for the development of enzyme- and microorganism-based biofuel cells because the NAD/NADH system can be served as redox mediator for many enzymes and redox-proteins, and in addition, NAD is a cofactor of NAD-dependent enzymes.

The application of MXenes for the design of catalytic sensors based on enzymes seems rather effective due to metallic conductivity of these compounds.

Ti_3_C_2_-based MXene, which was modified by Persian blue (PB), was applied in the design of “wearable” electrochemical biosensors [77] and showed good sensitivity to glucose and lactose applied MXene increased immobilization efficiency of immobilized enzyme, permeability of oxygen into biosensing structure, where it has taken part in the charge transfer from GOx. These sensors were integrated within flexible polymeric structures and used as wearable biosensing devices for the determination of lactose and glucose in actual concentration range of 1–20 mM with the sensitivity of 11.4 mA × mM^−1^ × 1cm^−2^ and 35.3 mA × mM^−1^cm^−2^, respectively.

Two types of screen-printed-electrodes: (i) urease, methylene blue (MB), and Ti_3_C_2_T_x_ based screen-printed-electrode (urease/MB/MXene/SPE); and (ii) Ti_3_C_2_T_x_ based screen-printed-electrode (MXene/SPE) were designed and applied in microfluidic electrochemical systems dedicated to continuous monitoring of urea and creatinine in whole blood were developed [78]. During the development of this biosensor enzyme, urease was immobilized using glutaraldehyde as a cross-linking agent, which binds enzymes well on electrode surfaces [58]. The first electrode based on urease/MB-MXene deposited on a screen printed electrode was used for the determination of urea in the range of 0.1–3 mM and the second one, MXene deposited on screen printed electrode, was used for the determination of creatinine in the range of 0.02–1.2 mM; in this electrode MXene was served as the electro-catalyst. These electrodes are incorporated within a microfluidic system and applied for the determination of urea and creatinine in whole blood. It is very remarkable that some MXenes involved in composite structures are exhibiting peroxidase-like activity and have been applied in the design of biosensors, for example, MXene-Ti_3_C_2_/CuS nanocomposite based sensor dedicated to colorimetric determination of cholesterol [79] and lactate dehydrogenase (LDH) based heterostructure of a MXene@NiFe-LDH for the detection of glutathione [80].

Nowadays some sensors are implantable; therefore, biocompatibility aspects of such sensors are very important. Here MXenes are especially welcome because some MXenes are biocompatible and almost non-toxic to some living cells such as mouse preosteoblast cell and mouse fibroblast cell lines [81] and mammalians such as mice [82]. However, despite some positive results, further investigations on biocompatibility of MXenes in both in vitro and in vivo toxicity assessment based on the determination of either reproductive toxicity, genotoxicity, or both, are needed before the design of implantable MXene-based biosensors and biofuel cells.

## 3. Direct Charge Transfer between Redox Proteins and MXenes

**Figure 2:**
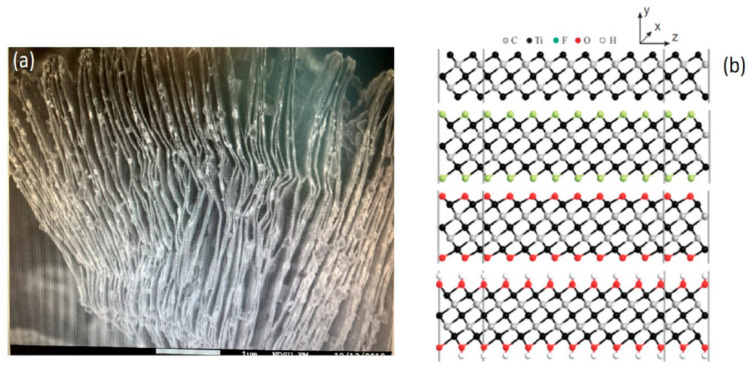
2D multi-layered Ti_3_C_2_ MXene sheets (**a**) scanning electron microscopy image; (**b**) pristine and surface-terminated Ti_3_C_2_ MXene with different functional groups. Reprinted from [86].

Direct charge transfer was observed in electrochemical biosensors based on Ti_3_C_2_ MXene deposited on glassy carbon electrode (GCE) and modified by immobilized hemoglobin (Hb) and Nafion in order to establish a Nafion/Hb/Ti_3_C_2_/MXene/GCE-based structure [22]. This sensor was suitable for the determination of nitrite in water samples. Catalytic activity of NO_2_^−^ reduction in this sensor [19] was based on proton-coupled reaction [83]. The other research group designed a mediator-free enzymatic electrochemical biosensor based on Ti_3_C_2_ MXene and immobilized hemoglobin and reported the sensitivity of this sensor towards hydrogen peroxide (H_2_O_2_), with linear range between 0.1 and 260 mM [22]; the authors speculated that this structure exhibits some ”organ-like” properties because it shows high efficiency towards reduction of hydrogen peroxide. This efficiency was achieved due to exfoliation of the MAX-phase, and it was determined that even layers that are thinner than 20 nm still show very high catalytic activity [22]. Exfoliation of separated sheets is observed each time when MXenes are formed [84,85], and there are some evidences that MXenes are providing both compatible environments for immobilized proteins where they retain catalytic activity, very large surface area (Figure 2a) and some functional groups, which can be used for the immobilization of enzymes (Figure 2b) [77].

It should be noted that hemoglobin (Hb) is a very suitable candidate for the development of cathodes for biofuel cells [32]; therefore, this MXene/Hb based electrode seems very pertinent for the design of biofuel cells. It was demonstrated that even better current density can be achieved if, instead of bare Ti_3_C_2_, a heterostructure based on TiO_2_-Ti_3_C_2_ nanocomposite is deposited on the GCE electrode and later it is modified by hemoglobin [22]. In this research, a designed Nafion/Hb/TiO_2_-Ti_3_C_2_-structure based biosensor was characterized by good sensitivity (of 447.3 mA × mM^−1^ × cm^−2^) towards H_2_O_2_ with a linear range of 0.1–380 mM, and LOD of 14 nM [22]. The Nafion/Hb/TiO_2_-Ti_3_C_2_/GCE biosensor [22] showed much better long-term stability in comparison with previously described Nafion/Hb/Ti_3_C_2_/GCE biosensors [22]. It seems that TiO_2_ significantly improves the biocompatibility and can advance the conductivity of the formed structure, especially if the nonstoichiometric form of titanium oxide TiO_2−x_/TiO_2_ is formed, which increases both conductivity and catalytic activity of formed heterostructures [87]. This effect is based on the fact that the TiO_2−x_/TiO_2_^−^ based structure has rather high concentrations of “oxygen vacancies”, which are responsible for n-type charge mobility in this semiconducting heterostructure [88]. Very recently, in one research conducted by our group, we predicted that such oxygen vacancies are providing increased sensitivity towards some reducing and oxidizing gases and VOCs [87]. These TiO_2_-based structures can be reduced and can form nonstoichiometric titanium oxides (TiO_2−x_), which can be partly based on Magnéli phases with stoichiometry of Ti_n_O_2n−1_ [89,90]. It was determined that in the TiO_2−x_ structure, which has a rather low “x” value varying between 0 and 0.10, therefore, so called “point defects” dominate in TiO_2−x_ crystal structure [91], and such structures possess high numbers of interstitials based on either Ti^3+^ and Ti^4+^, great ability of oxygen vacancies, or both. The concentration of above mentioned defects in the crystal structure of TiO_2−x_ is increased by increased “oxygen deficiency” rate. Some researches revealed that in Magnéli phases having rather high “x” values between 0.10 and 0.34 crystallographic shear planes are significantly extended [92]. These TiO_2-x_ heterostructures are stable, therefore, they are finding many applications in catalytic decontamination of waste-water and in the development of batteries and fuel cells [93,94]. Hence, there are some expectations that these physical properties of TiO_2−x_/TiO_2_-based heterostructures will improve performance of some MXene-based sensors and biosensors based on enzymes that are exhibiting direct charge transfer [40,41]. There are some indications that tungsten-based MXenes [95] can be advanced by the incorporation of either stoichiometric, nonstoichiometric tungsten oxide, or both [96,97], and will find some applications in the development of biofuel cells. The application of ink-jet printed MXene with graphene oxide heterocomposite (Ti_3_C_2_/GO) was also modified with Hb [23] and applied in biosensors for the determination of H_2_O_2_. Sensitivity of MXenes towards pH [98] can be well exploited in the design of some biosensors based on enzymes with pH that changes during catalytic action.

## 4. Affinity Sensors Based on MXenes Modified by Immobilized Affinity Agents

Affinity sensors are analytical devices, which specifically recognize analyte and form stabile complexes with analytes. According to applied analyte-binding, affinity sensors are classified into immunosensors, DNA-sensors, RNA-sensors and molecularly imprinted polymer based sensors. In some recent researches it was demonstrated that MXenes can be applied in the design of various affinity sensors. A very promising direction here is to design artificial biological recognitions systems based on molecularly imprinted polymers (MIPs) which were developed for proteins [99], DNA-based structures [100], but MIP-based sensors work especially well for the determination of small molecular weight analytes such as caffeine [101], theophylline [102], et cetera. In this research direction, hierarchical porous MXene/amino carbon nanotubes-based molecular imprinting sensor for the determination of low molecular mass analyte, fisetin, has also already been reported [103]. However, larger molecular mass analytes such as proteins were also determined by some MXene-based sensors based on immobilized receptors, for example, Ti_3_C_2_-based MXene was modified by biological receptor after the activation with 3-aminopropyl triethoxysilane (APTES) in order to perform covalent binding, and it was applied in the design of affinity sensor [104] for the determination of cancer biomarker, carcinoembryonic antigen [105]. In this research it was reported that the carboxylic group of anti-carcinoembryonic-antibodies binds well to the amino group of f-Ti_3_C_2_ MXene and forms a covalent bond. Hexaammineruthenium ([Ru(NH_3_)_6_]^3+^) was applied as a redox-probe for potentiodynamic measurement based determination of analytical signal. Authors declared extremely long linear detection range of this sensor, which was in the range from 10^−13^ to 2 × 10^−6^ ng/mL with great sensitivity of 37.9 mA ng/mL × cm^−2^ per one decade of concentration with extremely low LOD of 0.000018 ng/mL. This sensor operated well in human serum samples. Application of MXenes for the design of affinity sensors based on antibodies and some other affinity agents offers new avenues for the development of efficient affinity sensors. MXenes were applied for the design of chimeric DNA-functionalized sensors for mapping of some cancer biomarkers in living cells [106] and DNA-sensors suitable for the determination of label-free mismatches of DNA in real human samples [107]. RNA sensors based on the application of MXenes were also reported where a novel label-free electrochemical strategy for the determination of miRNA-182 detection based on MoS_2_/Ti_3_C_2_ nanohybrids was applied [108]. microRNA-155 detection based on AuNPs/Ti_3_C_2_ MXene three-dimensional nanocomposite for exonuclease III-aided cascade target recycling was designed [109] and oncomiRs detection based on synergetic signal amplification AuNPs/MXene were reported [110]. Very different application of DNA-based structures (such as DNA-aptamers) in the design of bio-recognition elements was applied in sensors dedicated to rapid electrochemical detection of thyroxine [111]. In another research, Ti_3_C_2_-based MXene was modified by DNA-aptamer applied in luminol-based chemo-luminescence based affinity sensor for the determination of MCF-7 exosomes [112], which established highly sensitive electro-generated chemo-luminescence. A glassy carbon electrode was modified by poly-nisopropylacrylamide/Au, which has provided a higher concentration of carboxyl available for covalent immobilization of DNA-aptamer, which has selectively recognized MCF-7 exosomes with LOD of 125 particles per mL^−1^. Moreover, a label-free electrochemical biosensor for highly sensitive detection of gliotoxin based on DNA nanostructure/MXene nanocomplexes was also reported [113].

**Figure 3:**
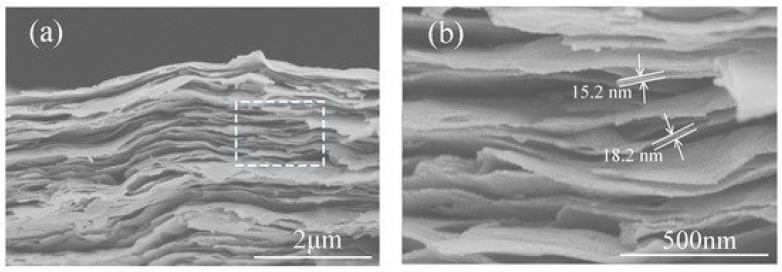
SEM images of (**a**) a cross-section of Ti_3_C_2_T_x_ film and (**b**) an enlarged part with estimated flake thickness. Reprinted from [114].

MXene-based structures are able to selectively adsorb different molecules through physical adsorption or electrostatic attraction, and lead to a measurable change in the conductivity of the material with high signal-to-noise ratio and excellent sensitivity (Figure 3). Therefore, sensors based on 2-dimensional Ti_3_C_2_ MXene-based nanosheets were characterized by good sensitivity and selectivity towards PGE2 and 8-HOA, which are both present in A549 lung cancer cells [86].

## 5. Non-Enzymatic Biosensors Cell Electrodes

Non-enzymatic biosensors and biofuel cell electrodes are electrochemical systems which are suitable for the determination of biological compounds and catalyze spontaneous oxidation/reduction of various biological compounds by generation of substantial potential (in the range of 50–1200 mV) and electrical current. Nonezymatic glucose sensors based on application of Ni-nanoparticle/polypyrrole composite was reported recently by our group [33]. The application of MXenes in non-enzymatic glucose sensors also seems very promising as it was demonstrated by the application of three-dimensional porous MXene/NiCo-LDH composite in the design of high performance non-enzymatic glucose sensors [115]. However, despite mentioned achievements, until recently most glucose oxidase (GOx) based sensors used are in this area [40,41,42,43,44,45,46], and during catalytic action of GOx as well as by action of many other oxidases hydrogen peroxide (H_2_O_2_) is produced. Some recent reports in this area illustrate that hydrogen peroxide can be easily determined by non-enzymatic PB/Ti_3_C_2_ hybrid nanocomposite [116]. 

In order to design other non-enzymatic sensors, MXene was introduced into graphite composite paste in order to design (MXene/GCPE)-electrodes, which were sensitive to adrenaline by chronoamperometry with LOD of 9.5 nM [117]. Very efficient determination of adrenaline, serotonin and ascorbic acid was achieved by differential pulse voltammetry (DPV), which enabled separate characteristic DPV-peaks for adrenaline, serotonin and ascorbic acid.

Screen-printed electrodes modified by Ti_3_C_2_T_x_ MXene modified (MXene/SPE) were applied for simultaneous detection of acetaminophen and isoniazid drugs [118]. DPV was applied as a detection method, which enabled distinguishing of characteristic DPV-peaks of acetaminophen and isoniazid with linear range between 0.25 and 2000 mM and LOD of 0.048 mM for acetaminophen and linear range between 0.1 and 4.6 mM and LOD of 0.064 mM for isoniazid.

Several MXene-based NH_3_ sensors were developed, where the remarkable properties of MXenes to adsorb gaseous materials were well exploited [119,120]. Moreover, catalytic properties of MXenes can be exploited for catalytic determination of various chemical and biochemical compounds [69].

## 6. Immobilization of Enzymes and Affinity-Agents on MXenes

Oriented immobilization is a very important issue in the design of enzymatic immunosensors and biofuel cells [32]. Well oriented enzymes exhibit sufficient activity, because in such cases the substrate has good access into active sites with lower diffusional limitations in comparison, when the enzyme’s active center is oriented upside down and oriented towards the electrode. The only exception is when the direct charge transfer is observed between electrode and enzyme, then the orientation is playing a different role; therefore, the active redox center should be oriented towards the electrode [40,41]. The advantage of MXenes in the application of biofuel cells would be useful for the adsorption of redox enzymes within 2D planes [121,122,123], because in such a system the orientation of enzymes would play a less critical role and in such a way MXene 2D planes will significantly increase the electrochemically active surface area of electrodes [3]. It is remarkable that MXenes are suitable to be applied for both anodic and cathodic potentials [124]. This property is highly appreciable for the development of biofuel cells because both electrodes can be based on very similar kinds of modifications and can be used for similar enzyme or cell immobilization procedures.

Rather unusual morphology and the ability of MXenes to be split into multiple one dimensional planes [125] (Figure 2 and Figure 3) during the preparation of these materials, enables “to load” MXene-based matrix by high concentrations of enzymes and microorganisms, which together with remarkable metallic conductivity of MXenes increases the applicability of these materials for the design of both enzymatic and microbial biofuel cells. It is remarkable that MXenes offer a good environment for immobilized enzymes or other proteins, which enables retaining sufficient catalytic activity [69,73]. However, up to now, only rather small sheets of MXenes were developed (up to maximum 1 µm in length and width), therefore, they can just be treated as deposits on other substrates.

**Figure 4:**
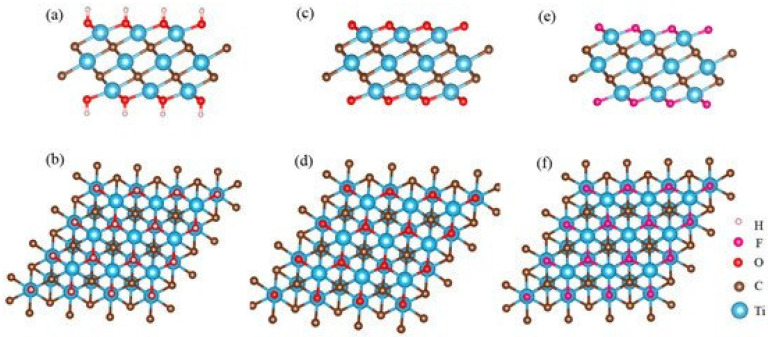
The structure of Ti_3_C_2_ nanosheets with different functional groups from side and top views: (**a**,**b**) Ti_3_C_2_(OH)_2_; (**c**,**d**) Ti_3_C_2_O_2_ and (**e**,**f**) Ti_3_C_2_F_2_. Reprinted from [133].

During the design of immunosensors and affinity sensors, the proper side of the affinity site of antibodies or other receptors should be oriented towards the solution with the analyte, which can be achieved by proper terminal groups of MXenes that are mostly suitable for covalent attachment [13,14,15]. Some affinity sensors have been designed by the immobilization of affinity-agents by simple adsorption on the surface of working electrode, which was modified by MXene-based sheets [31]. However, this strategy is not very efficient due to the random orientation of these affinity-agents, therefore, improved strategies, which enable orientation of antibodies should be adapted for the development of more efficient affinity sensors [126,127]. A number of such strategies were reported in a targeted review dedicated to the development of immunosensors based on oriented antibodies by Ramanaviciene and Makaraviciute [127] and in their experimental works it was well demonstrated that whole antibodies [128], receptors [129] and some particular parts of antibodies, which were chemically split into two or four pieces [126,127], can be immobilized in oriented-fashion on electrode surfaces. It should be noted that MXenes formed by chemical etching usually possess various surficial functional groups, mostly fluorine (−F) hydroxyl (−OH) or oxygen (−O) [130,131,132] (Figure 4). It is related to the chemical formula of MXenes, which is “Mn+1XnTx”, where T indicates surficial functional groups. Ti_3_C_2_ MXene formed by chemical etching can be based on the following three structures: Ti_3_C_2_(OH)_2_, Ti_3_C_2_O_2_ and Ti_3_C_2_F_2_ (Figure 4) [133]. The quantity and structure of terminal groups is mostly influenced by the synthesis protocol; therefore, the structure of terminal groups can be tailored, which is very important for the covalent immobilization of proteins (e.g., antigens or antibodies), which are used in immunosensor design as biological recognition parts [35].

Ti_3_C_2_-based MXene was applied in the design of biomimetic-sensors, which was based on adenosine triphosphate (ATP) deposited on MXene surface and then modified by Mn_3_(PO_4_)_2_; this system was applied for amperometric determination of superoxide anions (O_2_^−^) [134], which were generated by HepG2 cells. In this chemical sensor, Mn_3_(PO_4_)_2_ was sensing superoxide (O_2_^–^) in the range between 2.5 and 14 mM, sensitivity of 7.86 mA × mM^−1^ × cm^−2^ and LOD of 0.5 nM. In addition to amperometric measurements, capacitance measurements can also be applied due to high capacitance storage ability of MXenes; in this direction, Ti_3_C_2_ MXene nanosheet-based immunoassay with tyramine-enzyme repeating detection of prostate-specific antigens on interdigitated micro-comb electrodes was designed [135].

## 7. Conclusions and Future Trends

MXenes are 2D-materials, which show real potential for the application in the design of biosensors, biofuel cells and bioelectronics. MXenes are opening a new avenue for the development of conducting composites with metallic conductivity, which can advance sensing properties of amperometric enzymatic biosensors, because direct charge transfer between MXenes and heme-based redox proteins (hemoglobin) has already been reported. This finding opens a new avenue for the design of MXene-based biosensors and biofuel cells with other redox enzymes that are capable of direct charge transfer. Moreover, the advantage of MXenes in the application of biofuel cells would be their ability to adsorb redox enzymes within 2D planes (Figure 2), because in such a system the orientation of enzymes would play a less critical role, and in such way it will significantly increase electrochemically active surface areas of biofuel cell electrodes. The disadvantage of MXenes is that up now, only rather small sheets of MXenes were developed (up to maximum 1 µm in length and width).

Some terminal groups like −OH can be introduced into MXenes structures, which offers the possibility of immobilizing biological recognition exhibiting proteins in an oriented way; therefore, such ability can be well exploited for oriented immobilization of enzymes and antibodies. However, this property according to our best knowledge was never applied for the immobilization of biological recognition elements required for the design of enzymatic and affinity sensors.
